# Comparative Study of Hydroxytyrosol Acetate and Hydroxytyrosol in Activating Phase II Enzymes

**DOI:** 10.3390/antiox12101834

**Published:** 2023-10-07

**Authors:** Xuan Zou, Mengqi Zeng, Yuan Zheng, Adi Zheng, Li Cui, Wenli Cao, Xueqiang Wang, Jiankang Liu, Jie Xu, Zhihui Feng

**Affiliations:** 1 National & Local Joint Engineering Research Center of Biodiagnosis and Biotherapy, The Second Affiliated Hospital of Xi’an Jiaotong University, Xi’an 710004, China; zuseon@xjtu.edu.cn; 2 Precision Medical Institute, The Second Affiliated Hospital of Xi’an Jiaotong University, Xi’an 710004, China; 3 Frontier Institute of Science and Technology, Xi’an Jiaotong University, Xi’an 710049, China; 4 School of Health and Life Sciences, University of Health and Rehabilitation Sciences, Qingdao 266071, Chinaj.liu@xjtu.edu.cn (J.L.); 5 Department of Pediatrics, Central Hospital Affiliated to Shandong First Medical University, Jinan 250013, China; 6 School of Medicine, Northwest University, Xi’an 710069, China; 7 Center for Mitochondrial Biology and Medicine, The Key Laboratory of Biomedical Information Engineering of Ministry of Education, School of Life Science and Technology, Xi’an Jiaotong University, Xi’an 710049, China

**Keywords:** hydroxytyrosol acetate, nuclear factor E2-related factor 2, phase II enzymes, oxidative stress

## Abstract

Nuclear factor E2-related factor 2 (Nrf2) is fundamental to the maintenance of redox homeostasis within cells via the regulation of a series of phase II antioxidant enzymes. The unique olive-derived phenolic compound hydroxytyrosol (HT) is recognized as an Nrf2 activator, but knowledge of the HT derivative hydroxytyrosol acetate (HTac) on Nrf2 activation remains limited. In this study, we observed that an HT pretreatment could protect the cell viability, mitochondrial membrane potential, and redox homeostasis of ARPE-19 cells against a t-butyl hydroperoxide challenge at 50 μM. HTac exhibited similar benefits at 10 μM, indicating a more effective antioxidative capacity compared with HT. HTac consistently and more efficiently activated the expression of Nrf2-regulated phase II enzymes than HT. PI3K/Akt was the key pathway accounting for the beneficial effects of HTac in ARPE-19 cells. A further RNA-Seq analysis revealed that in addition to the consistent upregulation of phase II enzymes, the cells presented distinct expression profiles after HTac and HT treatments. This indicated that HTac could trigger a diverse cellular response despite its similar molecular structure to HT. The evidence in this study suggests that Nrf2 activation is the major cellular activity shared by HTac and HT, and HTac is more efficient at activating the Nrf2 system. This supports its potential future employment in various disease management strategies.

## 1. Introduction

With human lifespan extensions and demographic shifts, the proportion of people over 60 years old has increased. This has led to an urgent need for an effective therapeutic strategy for age-related diseases such as age-related macular degeneration (AMD). In mammalians, the macular region of the retina converges with the major photoreceptors; its degeneration causes central vision loss. AMD is a progressive degenerative disease of the macular region and is considered to be the leading cause of blindness in the elderly [1,2]. Retinal pigment epithelium (RPE) cells provide support for the survival and normal working of photoreceptors by performing several functions, including transporting nutrients and re-isomerizing visual pigment [3,4]. The RPE is susceptible to oxidative damage from intracellular free radicals or other external stimuli. It is generally believed that accumulated oxidative damage caused by aging is the main factor for RPE dysfunction, which contributes to AMD [5]. Recent studies have demonstrated that GSH depletion and tert-butyl hydroperoxide (t-BHP) incubation induce RPE ferroptosis, identifying ferroptosis as a major contributor to oxidative-stress-mediated RPE cell death [6,7,8].

Nuclear factor E2-related factor 2 (Nrf2) is a key regulator of phase II detoxification in response to oxidative/xenobiotic stress. In normal conditions, Nrf2 binds to the Kelch-like ECH-associating protein 1 (Keap1) in a low-activity state. Under oxidative stress, Keap1 is modified to promote Nrf2 release and translocation into the nucleus [9]. Nrf2 translocation drives the expression of downstream antioxidative enzymes, known as phase II enzymes, including cytoprotective gene heme oxygenase 1 (HO-1) and H:quinone oxidoreductase (NQO1) [10,11,12]. Heme oxygenase catalyzes the degradation of ferrous iron, carbon monoxide, and bilirubin [13]. NQO1 participates in the regulation of superoxide reductase activity and NAD+ generation [14]. Nrf2 promotes glutathione levels by regulating genes for glutathione metabolism such as the glutamate-cysteine ligase catalytic subunit (GCLC) and modifier subunit (GCLM) [15]. In vitro studies have demonstrated that Nrf2 plays an important role in oxidative-stress-induced RPE cell death [16,17,18]. Accumulating evidence has revealed that HO-1 is involved in RPE ferroptosis [8,19]. Therefore, the activation of Nrf2/HO-1 signaling could be a potential target mechanism for the protection of the RPE from oxidative stress, which could be beneficial to AMD.

Olive oil has abundant phenolic compounds. Among them, hydroxytyrosol (HT) is the most effective antioxidant [20]. In recent decades, understanding the beneficial role of HT in the aging process has achieved significant progress. HT has demonstrated a potential protective effect in several age-related diseases, including neurodegenerative diseases and AMD [21]. Hydroxytyrosol acetate (HTac), a natural derivative of HT, is a major component in olive phenols, but has received limited attention. The concentration of both HT and HTac is affected by the cultivar and processing methods of olive oil. HT concentrations range from 18 to 177 μM, whereas HTac concentrations are relative higher than HT and can range from 23 to 654 μM in different types of olive oil [22]. HTac has been reported to possess antioxidant and anti-inflammatory activities [23], presenting a greater bioactivity than HT in improving cognition [24]. The detailed mechanisms underlying HTac benefits remain largely unexplored. We previously observed that HT could protect RPE cells from oxidative stress by activating the Nrf2 signal pathway, thus improving mitochondrial biogenesis and the oxidation status [17,25,26]. Whether HTac can work as an Nrf2 activator in protecting RPE cell functions is still unknown. Despite sharing a similar chemical structure, the similarities and differences of cellular responses to HTac and HT are limited. These are interesting and important aspects of HTac that warrant an extensive investigation.

In this study, we used the human retinal pigment epithelial cell line ARPE-19 to elucidate the potential protective role of HTac. We performed a comparative analysis between HTac and HT on the cellular responses. The results demonstrated that the cells presented different expression profiles under HTac and HT treatments in addition to consistently activating the Nrf2 system. HTac demonstrated a better capacity than HT in activating Nrf2 and protecting ARPE-19 cells against oxidative challenges. This supports its potential use in various disease management approaches, including those for AMD.

## 2. Materials and Methods

### 2.1. Chemicals and Reagents

Hydroxytyrosol, hydroxytyrosol acetate, LY294002, PD98059, SP600125, and SB202190 were purchased from TOPSCIENCE (Shanghai, China). The tert-butyl hydroperoxide solution, MTT, and JC-1 were purchased from Sigma (St. Louis, MO, USA). The cell culture medium, H2DCF-DA, and MitoSOX TM Red Mitochondrial superoxide indicator were purchased from Life Technologies (San Diego, CA, USA). The TRIzol reagent, PrimeScript RT-PCR kit, and SYBR PremixExTaq II kit were purchased from Takara (Japan). Antibodies against GAPDH (5174) and histone H1 (41328) were purchased from Cell Signaling Technology (Danvers, MA, USA). The antibody against Nrf2 (62352) was purchased from Abcam (Cambridge, UK).

### 2.2. Cell Culture

Human retinal pigment epithelial cell line ARPE-19 was cultured in a DMEM/F12 medium with an HEPES buffer containing 10% fetal bovine serum, 56 mM sodium bicarbonate, 2 mM L-glutamine, 100 U/mL penicillin G sodium, and 100 μg/mL streptomycin sulfate. The cells were maintained at 37 °C in a humidified atmosphere with 5% CO_2_. The medium was changed every two days.

### 2.3. Cell Viability Assay

A cell viability assay was performed using the compound 3-(4,5-dimethyl-2-thiazolyl)-2,5-diphenyl-2-H-tetrazolium bromide (MTT). ARPE-19 cells were seeded in 96-well plates at a density of 2 × 10^4^ cells per well. After the treatment, 200 μL of a serum-free medium containing 5 mg/mL of the MTT solution was added and incubated for 3 h. The medium was then discarded and the cells were washed with PBS. This was followed by the addition of 200 μL DMSO to each well. The absorbance was measured at a wavelength of 490 nm using a microplate fluorometer (Fluoroskan Ascent; Thermo Fisher Scientific, Inc.,Vantaa, Finland).

### 2.4. Mitochondrial Membrane Potential Assay

The mitochondrial membrane potential (MMP) was analyzed using the lipophilic cationic probe 5,5′,6,6′-terachloro-1,1′,3,3′-tetraethyl-imidacarbocyanine iodide (JC-1). ARPE-19 cells were seeded in 96-well plates at a density of 2 × 10^4^ cells per well. After the treatment, the cells were incubated with a serum-free medium containing JC-1 for 1 h. The medium was then discarded and the cells were washed with PBS. The fluorescence was scanned using a microplate fluorometer (FlexStation 3, Molecular Device, San Jose, CA, USA) at a 488 nm excitation wavelength and 538 and 590 nm emission wavelengths, respectively. The red/green fluorescence intensity ratio reflected the MMP.

### 2.5. Reactive Oxygen Species Analysis

The total cellular reactive oxygen species were analyzed using H2DCF-DA. Mitochondrial superoxides were observed using a MitoSOXTM Red Mitochondrial superoxide indicator. Briefly, the cells were cultured at a density of 1 × 10^5^ cells per well in 6-well plates. After the treatment, the cells were incubated with 10 μM MitoSOXTM Red in a serum-free medium for 10 min or with 20 μM H2DCF-DA in a serum-free medium for 30 min. After washing with PBS, the cells were observed by laser scanning confocal microscopy (Zeiss, Jena, Germany).

### 2.6. Transcriptomics Analysis

ARPE-19 cells were collected in TRIzol (Invitrogen, 15596018) after the treatments and delivered to Novogene Co., Ltd., (Beijing, China) in dry ice for the RNA-Seq analysis. Briefly, mRNA was purified from the total RNA using poly-T oligo-attached magnetic beads. Reference genome and gene model annotation files were directly downloaded from the genome website. HTSeq v0.9.1 was used to count the numbers of reads mapped to each gene. Genes located by DESeq with an adjusted *p*-value of <0.05 were assigned as differentially expressed. A corrected *p*-value of 0.005 and a log2 (fold change) of 1 were set as the thresholds for significant differential expressions.

### 2.7. Real-Time Quantitative PCR

After the treatment, the total RNA of ARPE-19 cells were isolated using the TRIzol reagent, following the manufacturer’s protocol. The total RNA pellet was then washed with 75% ethanol and resuspended in DEPC water. The RNA was subjected to reverse transcription using a PrimeScript RT-PCR kit (Takara, Beijing, China). A quantitative real-time PCR analysis was conducted using a SYBR PremixExTaq II kit (Takara, Beijing, China). The data were normalized to the expression level of β-actin. The detailed primers are presented in Table S1 of the Supplementary Materials.

### 2.8. Western Blot Analysis

The cells were lysed using a Western blot and an IP lysis buffer (Beyotime, Shanghai, China). The lysates were homogenized on ice and centrifuged at 12,000× *g* for 15 min at 4 °C. The supernatants were collected and the concentrations were analyzed using a bicinchoninic acid (BCA) protein assay kit. Equal amounts (15 μg) of the protein samples were separated by 10% SDS-PAGE and transferred to pure nitrocellulose membranes blocked with 5% non-fat milk in a TBST buffer. The membranes were incubated with primary antibodies at 4 °C overnight, followed by incubation with secondary antibodies at room temperature for 1 h. Chemiluminescent detection was performed using an ECL Western blotting detection kit (Thermo Fisher, Rockford, IL, USA). The images were analyzed using Quantity One software V4.6.7 (Bio-Rad, Shanghai, China) for the density ratio of the target proteins relative to histone H1 or GAPDH.

### 2.9. Statistical Analysis

All cellular experiments were repeated at least three times. The data were presented as the mean ± SEM. All statistical analyses were performed using GraphPad software (Prism 9.0, Boston, MA, USA). The significance of the differences between two groups was assessed by an unpaired Student’s *t*-test. Multiple groups were analyzed using a one-way ANOVA with Duncan’s test. For all comparisons, the level of significance was set at *p* < 0.05.

## 3. Results

### 3.1. Effects of HTac and HT on Cell Survival

**Figure 1:**
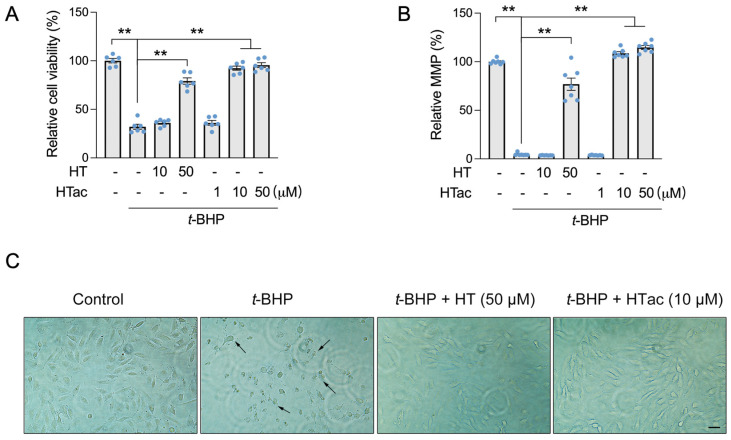
The effects of HTac and HT on cell survival. ARPE-19 cells were pretreated with HT at 10 and 50 μM and HTac at 1, 10, and 50 μM for 24 h, followed by 300 μM t-BHP treatment for another 24 h. Cell viability (**A**) and mitochondrial membrane potential (**B**) were analyzed. Cell morphology (**C**) was recorded using a microscope. Scale bar: 10 μm. The values are presented as the mean ± SEM and n = 6. ** *p* < 0.01 between the connected groups.

In our previous study, hydroxytyrosol (HT) was observed to protect ARPE-19 cells against t-BHP-induced mitochondrial dysfunction and cell viability loss at 100 μM [17]. In this study, we confirmed that an HT pretreatment at a dose of 50 μM demonstrated a significant protective effect against a t-BHP challenge. HT at a dose of 10 μM failed (Figure 1A,B). We observed that HTac presented a significant protective effect on the cell viability and mitochondrial membrane potential (MMP) at doses of 10 and 50 μM (Figure 1A,B). The protection was comparable between HTac at 10 μM and HT at 50 μM, which was further supported by cell morphology changes under the t-BHP challenge (Figure 1C). These data suggest that HTac had a larger capacity to protect ARPE-19 cells against oxidative challenges.

### 3.2. Effects of HTac and HT on Cell Oxidative Stress

**Figure 2:**
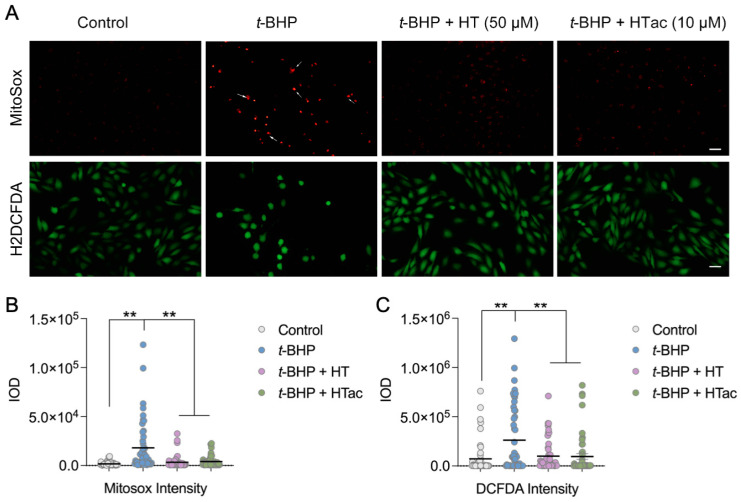
The effects of HTac and HT on cell oxidative stress. ARPE-19 cells were pretreated with HT at 50 μM and HTac at 10 μM for 24 h, followed by 300 μM t-BHP treatment for another 24 h. Levels of mitochondrial superoxides and cellular reactive oxygen species were analyzed by fluorescent confocal microscopy. (**A**) Microscopy image; scale bar: 10 μm. (**B**) Fluorescent intensity analysis of MitoSOX staining. (**C**) Fluorescent intensity analysis of DCF-DA staining. The values are presented as the mean ± SEM and n = 50. ** *p* < 0.01 between the connected groups.

It is well-acknowledged that t-BHP can be widely used as an alternative to hydrogen peroxide in oxidative stress studies. In addition to its oxidant properties, t-BHP can also trigger mitochondrial stress and dysfunction to promote cell death [27]. To further characterize the protective effect of HTac and HT against t-BHP-induced oxidative stress, the total cellular reactive oxygen species (ROS) were analyzed using H2DCF-DA and mitochondrial superoxides were observed using MitoSOX staining. As shown in Figure 2, a robust increase in mitochondrial superoxides was observed after the t-BHP treatment, whereas HTac at 10 μM and HT at 50 μM could both efficiently prevent mitochondrial stress (Figure 2A,B). Unlike MitoSOX staining, the DCF staining revealed a certain level of oxidants in the cultured cells under normal conditions, which was elevated after the t-BHP treatment (Figure 2A,B). Consistent with MitoSOX staining, pretreatments of HTac at 10 μM and HT at 50 μM significantly reduced the cellular ROS level (Figure 2A,B). These data indicate that HTac was more efficient than HT at reducing cellular and mitochondrial stress.

### 3.3. HTac Is More Efficient in Activating Phase II Enzymes Than HT

**Figure 3:**
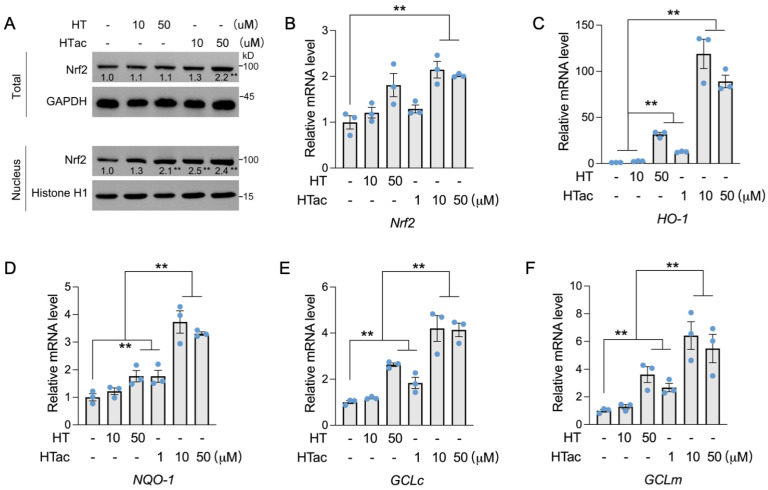
The effects of HTac and HT on the expression of phase II enzymes. (**A**) ARPE-19 cells were treated with HT at 10 and 50 μM and HTac at 1, 10, and 50 μM for 1 h. The total and nuclear Nrf2 protein levels were analyzed. ARPE-19 cells were treated with HT at 10 and 50 μM and HTac at 1, 10, and 50 μM for 24 h. The mRNA levels of Nrf2 (**B**), HO-1 (**C**), NQO-1 (**D**), GCLc (**E**), and GCLm (**F**) were analyzed. The values are presented as the mean ± SEM and n = 3. ** *p* < 0.01 between the connected groups.

Nrf2 has been well-demonstrated to regulate a series of endogenous antioxidative enzymes, known as phase II enzymes, including HO-1, NQO-1, and GCL [10,11,12]. The activation of phase II enzymes is suggested to be the major contributor to cellular protection against oxidative damage [28]. Herein, the effects of HTac and HT on the expression of Nrf2-mediated phase II enzymes were further examined. As shown in Figure 3, a short HT pretreatment at 50 μM had no effect on the total protein level of Nrf2, but could increase the nuclear location of Nrf2. Similar effects were also observed after the HTac treatment at 10 μM (Figure 3A). HTac at 50 μM increased both the total and nuclear Nrf2 protein levels (Figure 3A). An analysis of the prolonged treatment for 24 h indicated that only HTac could induce the mRNA expression of Nrf2 (Figure 3B). Consistent with nuclear localization, the target gene expression of Nrf2 (including HO-1, NQO-1, GCLc, and GCLm) was highly induced by HT at 50 μM and HTac at 10 μM and 50 μM (Figure 3C–F). The mRNA expression of these genes induced by HTac at 10 μM was significantly higher than HT at 50 μM (Figure 3C–F). These data suggest that HTac was more efficient at activating phase II enzymes than HT.

### 3.4. HTac Protects ARPE-19 Cells via the PI3K/Akt/Erk Pathway

**Figure 4:**
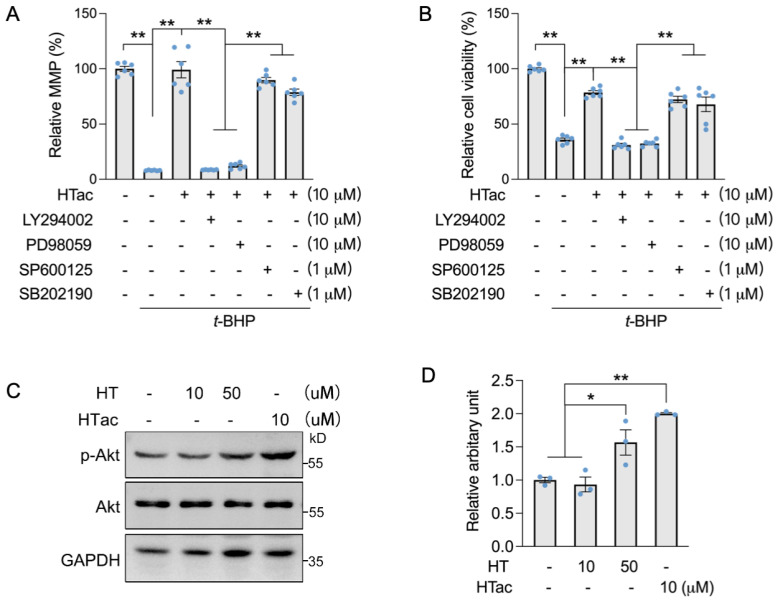
HTac protected cells against oxidative damage via the Akt/Erk pathway. ARPE-19 cells were treated with LY294002, PD98059, SP600125, and SB202190 1 h prior to HTac treatment at 10 μM for 24 h, followed by 300 μM t-BHP treatment for another 24 h. (**A**) Mitochondrial membrane potential and (**B**) cell viability were analyzed. ARPE-19 cells were treated with HT at 10, 50 μM, and HTac at 10 μM for 1 h, p-Akt level was analyzed by Western blot ((**C**) Western blot image; (**D**) arbitrary unit statistical analysis). The values are presented as the mean ± SEM and n = 3. ** *p* < 0.01, * *p* < 0.05 between the connected groups.

We previously demonstrated that HT could activate c-Jun N-terminal kinase (JNK) to promote the expression of Nrf2-mediated phase II enzymes for the protection of ARPE-19 cells against oxidative challenges [17]. Therefore, we used Akt and MAPK kinase inhibitors to confirm the major pathway that responded to HTac. Akt inhibitor LY294002, Erk inhibitor PD98059, JNK inhibitor SP600125, and p38 inhibitor SB202190 were incubated with ARPE-19 cells prior to the HTac treatment and following an oxidative challenge. An MMP assay confirmed that HTac could efficiently protect the cell mitochondrial membrane potential, which was significantly inhibited by LY294002 and PD98059 (Figure 4A). A similar effect was also observed for cellular viability (Figure 4B), suggesting that HTac could promote cell survival against oxidative challenges via the PI3K/Akt/Erk pathway. Moreover, short time treatment of HTac could significantly activate PI3K/Akt pathway at dose of 10 μM, while similar activation was achieved by HT at 50 μM (Figure 4C,D), further supporting the assumption of HTac having higher capacity than HT in activating phase II enzymes.

### 3.5. Cell Transcriptomics Analysis after HTac and HT Treatments

**Figure 5:**
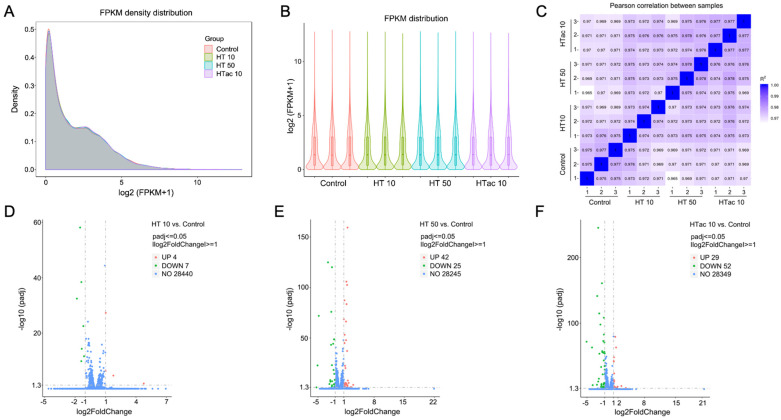
Cell transcriptomics response to HTac and HT treatments. ARPE-19 cells were treated with HT at 10 and 50 μM and HTac at 10 μM for 6 h. RNA-Seq analysis was performed to reveal cellular transcriptomics response. (**A**) The density plot of FPKM distribution with log2 (FPKM+1) on the horizontal axis and density on the vertical axis. (**B**) Violin diagram of FPKM distribution with each sample on the horizontal axis and log2 (FPKM+1) on the vertical axis. (**C**) Pearson correlation between samples. (**D**) Volcano plots of DEGs between HT 10 group and control group. (**E**) Volcano plots of DEGs between HT 50 group and control group. (**F**) Volcano plots of DEGs between HTac 10 group and control group. Control group: ARPE-19 cells without treatment; HT 10 group: HT treatment at 10 μM for 6 h; HT 50 group: HT treatment at 50 μM for 6 h; HTac 10 group: HTac treatment at 10 μM for 6 h; n = 3 for each group.

To further investigate the detailed transcriptomics response to HTac and HT treatments, RNA-Seq was employed after HTac and HT treatments for 6 h. The profile of the transcriptome sequence data are shown in Table 1. The four groups presented comparable raw and clean reads; all groups were perfectly mapped to human genomes, with uniquely mapped rates of over 90% (Table 1). The density and distribution of fragments per kilobase per million mapped reads (FPKM) at the transcriptome level among the four groups were detected (Figure 5A,B). The sample correlation tests revealed that there was clear heterogeneity among the four groups of twelve sequencing samples (Figure 5C). Together, the presented data indicated that all the sequencing samples were of a high quality and met the requirement of the subsequent analysis. In the following analysis of differentially expressed genes (DEGs), a fold change of ≥1 and padj ≤ 0.05 were used as screening thresholds. The volcano plots of the DEGs demonstrated that the HT treatment at 10 μM only identified 11 differentially expressed genes, including 4 upregulated genes and 7 downregulated genes (Figure 5D), whereas the HT treatment at 50 μM identified 67 differentially expressed genes, including 42 upregulated genes and 25 downregulated genes (Figure 5E). The HTac treatment at 10 μM presented 81 differentially expressed genes, including 29 upregulated genes and 52 downregulated genes (Figure 5F). The detailed gene information is provided in Table S2 of the Supplementary Materials.

### 3.6. HTac and HT Present Distinct Cellular Expression Profiles

**Figure 6:**
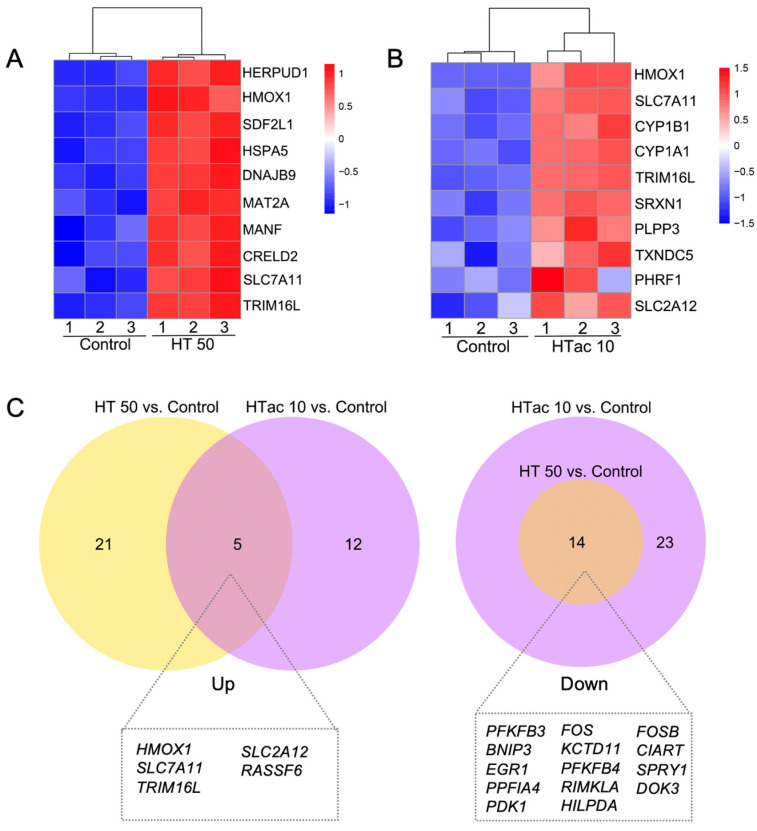
Comparison of HTac and HT gene expression profiles. (**A**) Heatmap of top 10 upregulated genes after HT treatment on ARPE-19 cells. (**B**) Heatmap of top 10 upregulated genes after HTac treatment on ARPE-19 cells. (**C**) Venn diagram of the identified DEGs. N = 3 for each group.

Both the HTac and HT treatments induced a limited gene expression at the transcriptome level. This could help to better understand the driving mechanisms underlying the various physiological benefits of HTac and HT. Figure 6 presents the heatmaps of the top ten upregulated protein-encoding genes (Figure 6A,B). The HT treatment at 50 μM significantly increased the expression of genes, including HERPUD1, HMOX1(HO-1), SDF2L1, HSPA5, DNAJB8, MAT2A, MANF, CRELD2, SLC7A11, and TRIM16L (Figure 6A), whereas HTac at 10 μM induced the expression of HMOX1(HO-1), SLC7A11, CYP1B1, CYP1A1, TRIM16L, SRXN1, PLPP3, TXNDC5, and SLC2A12 (Figure 6B). A further Venn diagram analysis revealed that 5 upregulated and 14 downregulated genes overlapped between the HTac and HT treatment groups (Figure 6C). Among the five upregulated genes, only HO-1 and SLC7A11 have been reported to be target genes of Nrf2 [29]. Whether SLC2A12, RASSF6, and TRIM16L are regulated by Nrf2 requires further investigation. Unlike the upregulated genes, the downregulated genes from the HT treatment all overlapped with the HTac treatment, which also presented another 23 downregulated genes that were not altered in the HT group (Figure 6C; Table S2). All these data indicated that although HTac and HT shared a similar molecular structure, they only presented a small overlapping profile at the transcriptome level. This included the expression of Nrf2-mediated phase II enzymes. The distinct expression profile of HTac compared with HT suggested that HTac may have unique physiological benefits.

## 4. Discussion

AMD is one of the leading causes of vision loss in the elderly. The dysfunction of RPE cells and the associated damage of retina photoreceptors are the major pathological factors leading to the progression of AMD [30]. Although multiple risk factors have been proposed to promote RPE dysfunction, an excessive production of ROS-associated oxidative stress has been presented as the leading mechanism. ROS-scavenging strategies have demonstrated consistent benefits in maintaining RPE functions [31]. Oxidative-stress-induced RPE cell damage has been well-used as a cellular model for the study of AMD pathology and target therapies.

The Nrf2 pathway is a primary endogenous antioxidative system employed by human cells, including the RPE, to fight against oxidative stress and maintain cellular redox homeostasis. It has been suggested that Nrf2 signaling is impaired in an aging RPE, which increases the risk of developing AMD [32]. The activation of Nrf2 could induce the expression of antioxidative enzymes such as HO-1, NQO-1, GLC, glutathione peroxidase, and glutathione transferase, the activation of which would increase the production of glutathione as well as the total cellular antioxidant capacity [33]. Therefore, Nrf2 has been well-recognized as a therapeutic target for the treatment of AMD. A series of natural compounds, including lipoic acid, lutein/zeaxanthin, and hydroxytyrosol (HT), has been implicated as being beneficial in the treatment of AMD via the targeting of Nrf2 activation [34].

HT is a well-known antioxidant and comprises 40% of the total phenolic compounds in olive oil, which is considered to be the symbol of a Mediterranean diet [35]. No genotoxicity of HT has been reported in humans, and multiple benefits have been revealed in the past decades through extensive studies [36]. We previously reported that HT could prevent diet-induced metabolic syndrome [37] and improve strenuous exercise-associated cardiac pathological changes [38] as well as protecting RPE cells against oxidative stress via activating Nrf2 signaling [17]. Other phenol compounds such as tyrosol and oleuropein have also been reported to activate Nrf2 for the protection of cellular stress and damage [39,40]. Hydroxytyrosol acetate (HTac), as a structural derivative of HT, is a polyphenolic compound present in olive oil. It has received limited attention in the research of Nrf2 signaling. HTac has been suggested to present neuronal and vascular endothelial cell protection as well as antibacterial activity in recent studies [23,24,41], but the effect of HTac on RPE cells remains unknown. Therefore, a comparative study of HTac and HT on RPE cells was performed. Consistent with a previous study [17], we confirmed that HT could protect mitochondrial functions and cell viability against t-BHP at a dose of 50 μM. HTac demonstrated a similar protection at a dose of 10 μM. Together with oxidant measurements and an Nrf2 target gene analysis, these data strongly support the notion that HTac is more efficient in activating phase II enzymes and protecting RPE cells than HT.

Nrf2 activity is regulated on multiple levels, including transcriptional, post-transcriptional, and protein-stability regulations. Keap1 is known to be the major binding protein affecting Nrf2 stability, which decreases under normal conditions because Keap1 can bind to Nrf2 for protein degradation. Under oxidative conditions, Keap1 is modified to promote Nrf2 release for the activation of phase II enzymes [9]. Other than Keap1, several kinases (including PI3K/Akt, ErK, p38, and JNK) have been reported to regulate Nrf2 protein stability and activity [42]. We previously demonstrated that HT could promote Nrf2 activity via the activation of the JNK pathway in ARPE-19 cells [17]. HT has also been reported to activate phase II enzymes via the Akt/Erk pathway in vascular endothelial cells [43]. Knowledge of the underlying kinase pathway that contributes to the induction of HTac on Nrf2 is limited. Here, we observed that unlike HT, HTac promoted cell survival against oxidative damage through the Akt/Erk pathway in ARPE-19 cells. The detailed mechanism requires further confirmation.

Although HTac shares the same main chemical structure as HT, they both appear to have distinct cellular transcriptome responses in addition to a small overlapping profile, based on our RNA-Seq analysis. This suggests that HTac has unique biological functions. Among the five upregulated genes that overlapped between the HTac and HT treatment groups, *HO-1* and *SLC7A11* are known to be regulated by Nrf2 and exert an antioxidative function [44]. Whether *SLC2A12*, *RASSF6*, and *TRIM16L* are the downstream targets of Nrf2 remains unknown. This is the first study to report the induction effect of HTac and HT on these three genes. SLC2A12 was previously identified as a glucose transporter; its overexpression improved insulin sensitivity in mice [45]. This further supports our previous study that identified the benefits of HT on diabetic mice [37] and suggests that HTac may also have beneficial effects on insulin-sensitivity improvements. RASSF6 and TRIM16L have been identified as tumor-suppressor proteins [46,47]. The increased expression of RASSF6 by HTac and HT treatments reveals a potential new mechanism of the antitumor effect of HT [48], encouraging the further study of HTac in tumor interventions. Apart from the overlapping upregulated genes, HTac uniquely upregulated the expression of *CYP1B1*, *CYP1A1*, *SRXN1*, *PLPP3*, *TXNDC5*, and *PHRF1*, which suggests the potential effect of HTac in detoxification, stem-cell differentiation, anti-inflammation, and DNA-damage repair [49,50,51]. The existence of uniquely downregulated genes from the HTac treatment also supports the speculation of distinct activities of HTac that warrant further investigation.

Our study demonstrated that HTac is a nutritional Nrf2 activator, presenting a more effective antioxidative capacity compared with HT in upregulating phase II enzymes and protecting ARPE-19 cells against oxidative challenges. In addition to an overlapping expression profile, HTac could also trigger a unique cellular response despite its similar molecular structure to HT. This suggests a greater number of potential bioactivities of HTac, which should be investigated to support its future employment in various disease management strategies.

## Figures and Tables

**Table 1:** Statistics for filtering and mapping reads.

Samples	Control-1	Control-2	Control-3	HT 10-1	HT 10-2	HT 10-3	HT 50-1	HT 50-2	HT 50-3	HTac 10-1	HTac 10-2	HTac 10-3
Raw reads	47939032	47880000	45618428	48772208	47614586	46835738	45019930	46509670	49756022	44914942	52660428	47572294
Clean reads	46431720	45692016	44196080	46586116	45334752	45742806	43542056	44870870	48029950	43278854	51208532	46256580
Q20 (%)	97.74	97.97	97.88	97.88	97.93	96.87	97.66	97.86	97.84	97.73	97.87	97.85
Q30 (%)	93.71	94.23	94.08	94.03	94.13	91.5	93.51	94	93.94	93.59	94.04	94
GC content (%)	50.21	50.08	50.29	50.23	50.13	49.6	50.09	50.13	49.96	50.24	50.37	50.35
Total map	44922259	44325313	42804025	45122822	43867105	43618780	42039485	43408363	46436530	41849147	49536077	44737898
	(96.75%)	(97.01%)	(96.85%)	(96.86%)	(96.76%)	(95.36%)	(96.55%)	(96.74%)	(96.68%)	(96.7%)	(96.73%)	(96.72%)
Unique map	42413515	45692016	42413515	42524604	41364784	41241266	39632293	40947247	43830529	39526822	46718527	42265694
	(91.35%)	(91.59%)	(91.59%)	(91.28%)	(91.24%)	(90.16%)	(91.02%)	(91.26%)	(91.26%)	(91.33%)	(91.23%)	(91.37%)

## Data Availability

The data presented in this study are available in the article.

## Supplementary Materials

The following supporting information can be downloaded at: https://www.mdpi.com/article/10.3390/antiox12101834/s1. Table S1: Detailed primer information; Table S2: Detailed information of DEGs.
